# Sudden Infant Death Syndrome (SIDS): State of the Art and Future Directions

**DOI:** 10.7150/ijms.89490

**Published:** 2024-03-25

**Authors:** Oscar Fraile-Martinez, Cielo García-Montero, Sofía Castellanos Díez, Coral Bravo, María de Guadalupe Quintana-Coronado, Laura Lopez-Gonzalez, Silvestra Barrena-Blázquez, Natalio García-Honduvilla, Juan A. De León-Luis, Sonia Rodriguez-Martín, Miguel A Saez, Melchor Alvarez-Mon, Raul Diaz-Pedrero, Miguel A Ortega

**Affiliations:** 1 Department of Medicine and Medical Specialities, Faculty of Medicine and Health Sciences, University of Alcalá, 28801 Alcalá de Henares, Spain.; 2 Ramón y Cajal Institute of Sanitary Research (IRYCIS), 28034 Madrid, Spain.; 3 Department of Public and Maternal and Child Health, School of Medicine, Complutense University of Madrid, 28040 Madrid, Spain.; 4 Department of Obstetrics and Gynecology, University Hospital Gregorio Marañón, 28009 Madrid, Spain.; 5 Health Research Institute Gregorio Marañón, 28009 Madrid, Spain.; 6 Department of Surgery, Medical and Social Sciences, Faculty of Medicine and Health Sciences, University of Alcalá, 28801 Alcala de Henares, Spain.; 7 Department of General and Digestive Surgery, University Hospital Príncipe de Asturias, 28805 Madrid, Spain.; 8 Department of Nursing and Physiotherapy, Faculty of Medicine and Health Sciences, University of Alcalá, Alcalá de Henares, Spain.; 9 Service of Pediatric, Hospital Universitario Principe de Asturias, 28801 Alcalá de Henares, Spain.; 10 Pathological Anatomy Service, Central University Hospital of Defence-UAH Madrid, 28801 Alcala de Henares, Spain.; 11 Immune System Diseases-Rheumatology and Internal Medicine Service, University Hospital Príncipe de Asturias, CIBEREHD, 28806 Alcalá de Henares, Spain.

**Keywords:** sudden infant death syndrome (SIDS), forensic findings, risk factors, preventive measures, etiopathogenesis

## Abstract

Sudden infant death syndrome (SIDS) is a type of death that occurs suddenly and without any apparent explanation, affecting infants between 28 days of life and up to a year. Recognition of this entity includes performing an autopsy to determine if there is another explanation for the event and performing both an external and internal examination of the different tissues to search for possible histopathological findings. Despite the relative success of awareness campaigns and the implementation of prevention measures, SIDS still represents one of the leading causes of death among infants worldwide. In addition, although the development of different techniques has made it possible to make significant progress in the characterization of the etiopathogenic mechanisms underlying SIDS, there are still many unknowns to be resolved in this regard and the integrative consideration of this syndrome represents an enormous challenge to face both from a point of view scientific and medical view as humanitarian. For all these reasons, this paper aims to summarize the most relevant current knowledge of SIDS, exploring from the base the characterization and recognition of this condition, its forensic findings, its risk factors, and the main prevention measures to be implemented. Likewise, an attempt will be made to analyze the causes and pathological mechanisms associated with SIDS, as well as potential approaches and future paths that must be followed to reduce the impact of this condition.

## 1. Introduction

Sudden infant death syndrome (SIDS) is an entity first defined in 1969 as a special subgroup of sudden unexpected infant death (SUID) that occurs in the postnatal period and shares certain epidemiological, clinical, and pathological features 1. According to the San Diego definition, SIDS is recognized as “the sudden unexpected death of an infant <1 year of age, with the onset of the fatal episode occurring during sleep, that remains unexplained after a thorough investigation, including the performance of a complete autopsy and review of the circumstances of death and the clinical history” 2. Previously, any death that occurred unexpectedly was classified as SIDS since, due to the lack of proper investigation, no explanation was found for the event. Thanks to the improvement of police investigation and autopsies, it was determined that not all deaths were unexplained, leading to the distinction between SUID and SIDS 3.

In 2017, SIDS was the fourth-leading cause of death among infants in the United States 4. Although this ranking has remained constant in successive years, the percentage of deaths due to SIDS tends to be lower each year 5. In Europe, a similar result has been observed in 14 different countries. From 2005 to 2015, both SUID and SIDS became rarer in several European countries, including Spain. Deaths from any of these circumstances accounted for 9.7% of all child deaths in this period 6. In Spain, SUID ranked third in the most frequent causes of infant death, thanks largely to the implementation of awareness campaigns 6.

Despite the reduction in the total number of cases and the success of these campaigns in some countries, many infants still die from SUID and SIDS. Even so, the impact of this type of event on families and the limited knowledge of the aetiopathogenesis of SIDS show the need to better understand this condition and the need to engage in advocacy to raise awareness as effective measures for its prevention. Thus, the present review aims to precisely describe SIDS and the mechanisms underlying this condition, as well as its main risk factors, the impact it has on affected families, and prevention measures specifically directed at this population. Similarly, new potential approaches will be considered that could help reduce the global impact of this condition.

## 2. Description, subcategories and forensic clues of SIDS

To certify the death of the infant and be able to classify it as SIDS, an autopsy must be done. A thorough investigation should also be conducted, and this should include examining the scene of the death, ideally with doll reenactment, documenting the circumstances of the death, reviewing the infant´s medical history, performing a radiographic examination, and driving a full autopsy with lab testing including histology, neuropathology, toxicology, and microbiologic studies 7. To date, however, despite the existence of established protocols, a great heterogeneity in the comprehensiveness of SIDS investigations exists in the United States 8. Accordingly, four major subcategories of SIDs can be recognized: 1) Category IA SIDS (classic features with complete investigation); 2) Category IB SIDS (classic features with incomplete investigation); 3) Category II SIDS and; 4) USID (unclassified sudden infant deaths), whereas some experts also include “temporarily interrupted SIDS” in cases of infants, which are resuscitated in extremis but later die 9. Table 1 collects the main features of the subcategories of SIDS, following the definitions given in the literature 2. However, later studies claim a need for reconsidering this type of classification, as some difficulties existed around the proposed definitions of each subcategory 10.

Regarding autopsy, multiple external and internal findings have been reported, but they are insufficient to explain the cause of death 11. Apart from autopsy, it would be of great aid to perform histopathological analysis, as well as to collect metabolic, radiological, microbiological and virological information. Additionally, it is necessary to take into account certain natural processes, such as putrefaction or autolysis, which can alter the results of the examination 12. Externally, the main orifices should be examined, such as the mouth, nose, ears, and the urogenital and anal orifices. This exam is done to determine if there are injuries and, if so, to know how they occurred. What must also be looked at is the lividity that the body can present to determine the true position in which the infant was at the time of death 13. Other external findings may include frothy, blood-tinged fluid at the nares in an otherwise well-developed infant 11. Apart from that, externally in this type of death, no injuries of any kind are found, except those that may have been caused by resuscitation attempts, which can be seen in some of the aforementioned orifices or on the skin 14. As it will be subsequently discussed, the posture of death is another important finding to consider, particularly if the baby is found lying face-down (prone position) 15.

Regarding the internal study, different organs and systems are particularly affected such as the respiratory system, heart, and central nervous system. Also, increased weights of different organs such as the thymus, lungs, liver, and brain appear to be observed in SIDS cases 16. In parallel, the internal examination should also be directed to confirm whether the lesions found in the external examination, which could have been described as resuscitation marks, are truly the product of resuscitation or could have been produced in another way. Internal examination should also consider possible congenital abnormalities of the cardiac conduction and the autonomic nervous system, mainly cardiorespiratory, of the first upper-digestive pathways, and arousal 17. Next, it is necessary to study whether there is possible sepsis in the infant. Infections in infants are usually caused by bacteria, which usually affect the lungs, an organ in which the pathologist can tell if there is a bacterial infection, though sepsis can necrotize and cause bleeding or atrophy of other organs, such as the liver and kidneys 12. Previous works have also found the relevance of extramedullary hematopoiesis in the liver of infants with SIDS secondary to anemia associated with intrauterine hypoxia or infections 18. Likewise, the bladder and rectum are two organs typically found empty, possibly reflecting an agonal event, or any type of shock 19.

Regarding specific clues observed in the respiratory system, intrathoracic petechial hemorrhages are common marks specifically observed in the thymus, pleura, viscera, and epicardium 20. Other internal observations include subacute inflammation of the upper respiratory tract, and pulmonary congestion/edema 11. It is necessary to determine the presence of infections in the most affected tissues, such as the epiglottis, trachea, respiratory tract, and bronchi. In them, a high percentage of immune cells should be observed as a response to the infection, and in some regions, there will be edema as a result of the infiltration 21. Something similar can be observed in cases of asthma, but these also present thickening in certain tissues. The same will be observed in the tissue extracted from the lungs, which may also present hemorrhages, which in some cases is consistent with resuscitation attempts and not with a possible infection. Death by aspiration is easily detectable since the lesions in the tissues would lack infiltrates. The same would happen if the infant has diseases associated with the development of the lungs, which can be seen to be different from normally developed lungs 12.

The study of other organs like the heart is also of great aid to studying the SIDS. In this case, it has to be seen if the infant may have suffered from myocarditis, which can be caused by an infection or by failures in the immune system and is characterized by myocardial inflammation, which can lead to arrhythmias 22. If it was caused by an infection, it would be lymphocytic myocarditis caused by a virus that would generate an infiltrate of lymphocytes 23. Instead, eosinophilic myocarditis could also be possible if the infiltrate is represented by eosinophils 24. Also, liquid, unclothed blood within the chambers of the heart is a common and almost consistent finding in SIDS 16.

It is important to study the central nervous system (CNS). The pathologist often looks for subdural hematomas, which are hemorrhages that occur on the brain surface in response to strong blows and that can be dated to know when they occurred 25. A study of the white matter of the brain should be done to determine whether it presents periventricular leukomalacia, a pathology that causes the intensity of circulation through the white matter to increase, producing nerve failures 26. Another white matter pathology that has been observed in some cases of SIDS is subcortical leukomalacia, which produces spongy changes, as well as inflammation in the axons, causing disruptions in nerve responses 12. In these same cases, it has been possible to see certain deformations in the hippocampus, which are not seen if the infant has epilepsy. The pathologist also tries to find any infections, which will produce infiltrates of different immune cells depending on the pathogen. Two such infections are meningitis and encephalitis 12.

**Figure 1:**
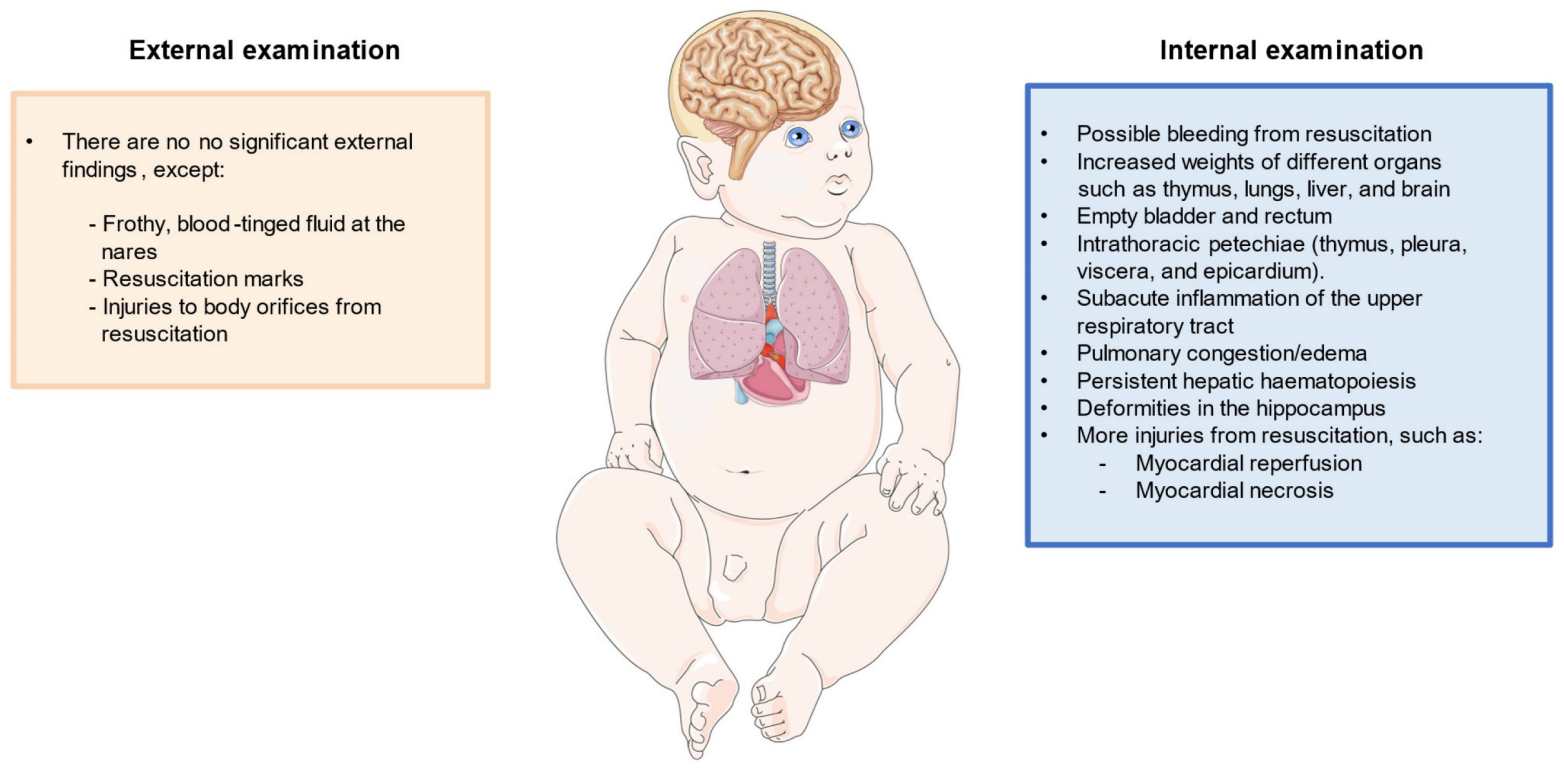
Representation of the most relevant histopathological findings described in an infant with SIDS.

Once the autopsy has been completed by studying the different tissues, a conclusion can be reached about the cause of death. If the infant showed signs of severe pathologies that might explain the death, e.g. infections, injuries, or malöformations, then the death could be attributed to a specific death circumstance, but if the autopsy does not reveal something specific, only resuscitation lesions have been found, and there are some characteristic signs of sudden death, then it will be classified as SIDS. *Figure 1* summarizes the most important histopathological findings that should be considered in the diagnosis of SIDS.

## 3. Risk factors

SIDS is made more likely by certain factors, which together are known as the "triple risk model". Within this model, three factors that lead to SIDS coexist and interact: the vulnerability of the infant, the critical period of development the infant is in, and exposure to external stressors that the baby cannot cope with 27.

Recognizing the vulnerability of the infant represents a real challenge since this vulnerability has been poorly defined 27. Some risk factors that can be related to this vulnerability have been recognized, such as some genetic polymorphisms, brain malformations, exposure to certain toxicological agents during pregnancy, and some complications during pregnancy 28. Similarly, it has been observed that male infants have a higher percentage of suffering from both SIDS and SUID 29. Another individual characteristic that can predispose the infant to SIDS is a low birth weight, generally less than 2.5 kg, or premature birth, defined as before 37 weeks of gestation 30. Ethnicity can also be related to the incidence of SUID/SIDS, although this relationship should be explored in greater depth 31. Finally, such factors as the mother's age and her educational level also seem to influence the risk of SIDS 30.

The critical period of the infant generally comprises the period between 2 and 4 months, since 90% of SIDS cases occur before the infant reaches 6 months 32. At this stage, the baby undergoes significant changes in the respiratory, circulatory, and autonomic nervous systems (ANS). The ANS is responsible for controlling the proper functioning of the organs so that any problem in the development of this system can affect the respiratory and circulatory systems.

As for external stressors, a wide variety of risk factors related to the environment have been described, mainly having to do with the sleep or feeding of the infant 33. Among the factors related to sleep, one of the most important is the position of the infant. If the infant is lying in the prone or lateral decubitus position, there is an increased risk of death from SIDS 33. Another factor that can increase death from this syndrome is the use of soft mattresses or pillows. If the mattress or sleeping surface is soft, and the infant sleeps in a prone position, it can lead to the formation of a pocket of carbon dioxide around the face, hindering the proper exchange of gases 15. Likewise, the potential ingestion of bacteria from contaminated sleep surfaces also explains the increased risk of SIDS in babies who are put to sleep prone 34. Indeed, compelling evidence seems to support the benefits of sleeping in a supine position and the back-to-sleep campaign (BTSC) 35. However, other authors have claimed that this association is not clear and that the correlation does not imply causality 36, thus denoting the need for further investigations on this important risk factor. Another risk associated with sleep is when the infant is overheated during sleep, which increases the risk of SIDS. The last of the sleep-related factors is that the infant has a bed with an adult, animal, or other object during sleep 37. In addition, leaving the infant sitting in an unusual place, such as the seat of a car or the arms of a sofa, apart from posing a risk of falling, also increases the risk of SIDS 28. As a feeding-related risk factor, formula-fed infants have been reported to have a higher risk of SIDS than those who are breastfed 38.

Overall, the triple risk hypothesis has long been the cornerstone of SIDS research for years. However, this hypothesis faces challenges as it suggests a causal role for the prone sleep position, despite instances of SIDS occurring in supine and side positions. This discrepancy indicates the need to reconsider the role of the prone sleep position as a direct cause and instead explore its potential to increase SIDS risk through different mechanisms like infections (see section 5).

## 4. SIDS prevention

As mentioned in the previous section, the risk of dying from this syndrome can be increased by certain risk factors, but certain recommendations reduce its risk.

One of the main ways in which the risk of SIDS can be reduced is by placing the infant in the correct position, the supine position. This position lowers the likelihood of death while sleeping and does not increase the risk of death by asphyxia or aspiration. On the other hand, although the prone position is not advisable for sleeping, "tummy time" is recommended. This expression means laying the child in the prone position, as long as the child is awake and supervised by the parents 39. Applying this technique for a time improves the infant's posture and strengthens the shoulders. Both sleep and tummy time should be done on firm surfaces. These surfaces must be free of any object, that is, the infant must sleep without any type of object around, such as pillows, toys, necklaces, or blankets that are not tight to the mattress 40. On the other hand, despite the recommendation not to sleep with objects, animals, or relatives, sleeping in the same room as the parents reduces the risk of SIDS.

Another aspect to take into account during sleep is not to wrap the infant too warmly. It is recommended not to put more than one extra layer beyond what an adult would wear when it is cold; if more layers are added, the infant has a greater risk of death. Similarly, the use of a pacifier when sleeping decreases the risk of SIDS 37. Breastfeeding during the first 6 months of life is also a measure supported by scientific evidence 41. Exclusive breastfeeding reduces this risk even more than if it is done in combination with bottles, even if the milk is always from the mother.

All these recommendations are arranged into different categories, ranging from those highly recommended by the United States Preventive Services Task Force (USPSTF) to those for which it cannot be known whether they are beneficial because there is not enough evidence 40. Table 2 summarizes the main measures and their level of evidence for preventing SIDS.

The creation of different programs and campaigns where these recommendations are explained has managed to significantly reduce cases of SIDS. One of these programs is the "Back to Sleep program". This campaign was created in 1994 by the American Academy of Paediatrics. It pushes for safe sleep practices (SSPs). These practices correspond to the above recommendations. Within these recommendations are three main ones, known as "ABC". This name comes from the acronym in English of the crucial practices to avoid SIDS, which are that the infant sleeps alone with nothing nearby; sleeping on the back, and sleeping in a crib 42. This program was aimed at parents to determine what practices they should carry out and which ones they should not. It was observed that it was also essential that neonatal staff apply it in the hospital since the nursing team would sometimes carry out certain practices contrary to recommendations, so that when the family got back home, the parents would imitate more the practices followed in the hospital than the published recommendations. For this reason, a common practice script was created for both hospitals and home, which specified when the recommendations should begin to be applied since they are not always applied from birth. This is because infants who are born prematurely do not receive SSPs at first. After all, it is recommended to lay them in the prone position to improve their respiratory action 42.

It is for all these reasons that the nursing team and parents must be trained correctly on why to carry out certain actions and when are the right times to do them. It is also important to monitor the habits of parents to see if they are following the recommendations. Before this campaign began, a high percentage of babies died from SIDS in the United States, which was reduced after the campaign was launched. Something similar happened in Spain, where there was a high percentage of sudden death that decreased when various campaigns were promoted during the 1990s. An increase in cases has been observed again, which indicates that these campaigns, carried out years back, should be promoted again to be sure families are educated about the risk that certain actions may bring 43.

Although training of parents and medical staff is important, training is also important for investigating the death of an infant. The United States Centers for Disease Control and Prevention created standardized guidelines on how to conduct a forensic investigation in which death was thought to have been caused by SUID or SIDS. Through all the information collected, the aim was to improve the process of classification of death through autopsy and to know with more certainty what circumstances had contributed to the death. All of this helped with the creation of education programs for safe sleep 29.

## 5. Aetiopathogenesis

One of the most challenging aspects of SIDS to clarify is the underlying aetiopathogenic mechanisms that explain the sudden death of the infant. These mechanisms are directly related to the risk factors the baby has been exposed to and that usually affect different organs and systems. The accumulated evidence shows that these alterations may have a genetic component, but the existence of other factors not related to the developmental pathologies of the infant, such as an infection, changes in metabolism, or inflammatory mechanisms, can increase the risk of SIDS 14. In this section, the main mechanisms potentially involved in the occurrence of SIDS will be summarized.

### a. Alterations in the central nervous system

Changes in CNS are thought to be critically involved in the aetiopathogenesis of SIDS, given its importance in the control and functioning of the organs and tissues of the body 44. However, it is unclear whether these changes are primary or secondary to the intricate pathogenic context related to SIDS. In 1990 Oehmichen outlined the multiple challenges of neuropathological research in SIDS, highlighting the absence of a definitive pathogenic concept due to divergent findings, and methodologic and interpretative problems 45. Nowadays, more than three decades later, the understanding of the CNS involvement in SIDS remains unclear, as some evidence supports that changes observed in the brain and other encephalic structures are somehow related to this entity.

Previous studies have shown the presence of neurological damage such as astrogliosis in the brainstem of infants who died from SIDS 46,47. Astrogliosis is the process by which astrocytes undergo some type of change caused by damage, disease, or injury of the CNS 48 These changes can be diverse and can manifest in different ways. On the one hand, transcriptomic and histopathological alterations can occur in these cells, including hypertrophy and proliferation phenomena, which can be associated with different mechanisms of neurological damage 49. Astrocytes are found throughout the CNS, including in the brain stem, which is responsible for controlling respiratory and autonomous processes, among others. Specifically, within this structure is the pre-Bötzinger (PBC) complex, responsible for the control of respiratory function 50. The functioning of this structure depends on the direct interaction of excitatory and inhibitory interneurons that, being complementary, cause the periodic rhythm of respiration to occur. Astrocytes also play a fundamental supporting role in this process, modulating homeostasis and neurotransmitter levels. Astrogliosis can therefore hinder the regulation of the PBC, leading to altered autonomic responses and thus respiratory failure 51. This failure includes a reduction in the supply of oxygen to tissues (hypoxia); an excess of CO_2_ in the blood (hypercapnia); or suffocation, which occurs when the airway is blocked. Some authors hypothesize that if there is a problem in the system, the defense mechanisms that infants engage in the face of any of these respiratory failures will not work correctly; that is, during one of these events, the baby may cry, open the eyes, or perform similar actions that put the body on alert, but as there is a defect, these corrective mechanisms will not be activated, and the baby can die anyway 14. Despite the involvement of astrogliosis in this process, it is not known whether it arises due to a neuropathological event of primary abnormal development or is secondary to hypoxic-ischaemic phenomena 52.

The neurotransmitters that astrocytes detect and that exist in the PBC are glutamate, gamma-aminobutyric acid, and dopamine, although the most relevant are serotonin and substance P 29. According to the literature, both serotonin and substance P are critically involved in the respiratory and cardiorespiratory function through the modulation of the PBC and raphe nucleus 53. In deaths attributed to SIDS, infants have presented a disorder in the development of the serotonergic system, specifically in the serotonin receptors, expressed in the brainstem 54. Previous research hypothesizes that these defects in the serotoninergic system originate prenatally and despite the precise causes of this dysregulation being unknown, it seems that is responsible for making infants more vulnerable to other factors involved in SIDS 55. Because of this, the search for promising biomarkers to identify the vulnerability and possible defects of infants is critically required. In this sense, Haynes et al. 56 found that serum serotonin levels in a subset (31%) of SIDS infants were elevated when compared with control infants. Thus, they proposed that peripheral serotonin levels could represent a potential biomarker for identifying vulnerable children, although further studies are warranted.

On the other hand, Substance P is a neuropeptide related to internal homeostasis, specifically the regulation of the respiratory rhythm, as well as cardiovascular control. It works together with serotonin when they are colocalized. Being related to the serotonin receptors, substance P can compensate for the failure of the serotoninergic system if the latter fails. In some cases, the damage is intensified if the substance P receptor has some type of abnormality, which is usually due to the close relationship between it and serotonin. The protein in charge of detecting substance P is the tachykinin receptor, specifically NK1, both of which are distributed throughout the CNS 57.

Finally, other works have suggested the role of some genetic variants involved in the development of the autonomic nervous system in infants that deceased by SIDS, as is the case of the gene PHOX2B 58, a gene involved in Congenital central hypoventilation syndrome (CCHS) 59. However, other studies have failed to find any significant association between this gene and SIDS 60,61, whereas it is still inconclusive whether different polymorphisms in PHOX2A, RET, ECE1, TLX3, and EN1 genes might be implicated in the autonomic dysfunction observed in SIDS neonates 62.

### b. Heart abnormalities

SIDS babies can also have abnormalities in the heart. Among these anomalies is long QT Syndrome (LQTS). In LQTS, the QT interval on the electrocardiogram is longer than normal, which causes lethal arrhythmias. The QT interval is the time it takes between ventricular depolarization (QRS complex) and repolarization (T wave) 63. This pathology is related to several mutations in the genes that code for cardiac ion channels, so it is hereditary. These channels are essential for the transmission of the heart pulse. A failure in one of them can interrupt the pulse, causing irregular heart rhythms. Among these genes, the two most relevant codes are for a sodium channel and a potassium channel. These are the *SCN5A* and *KCNQ1* genes, respectively, although it has been seen that the potassium channel can be encoded by other genes 64. Overall, the estimated percentage of genetic mutations affecting the heart in neonates with SIDS is estimated to be around 10% of cases 65.

Other cardiac pathologies can cause SIDS, such as catecholaminergic polymorphic ventricular tachycardia. This is a pathology similar to LQTS, but it is caused by the calcium channel gene *RYR2* or, less often, the sarcoplasmic reticulum protein calsequestrin 2 gene *CASQ2*. The mutation of the *RYR2* gene destabilizes the calcium channel by causing leakage of a large amount of calcium, which hinders the sodium-calcium exchange that helps generate the cardiac potential needed for the heart to function 66. Therefore, failures in this complex can cause fatal arrhythmias, as occurs with LQTS.

Other mutations associated with heart problems are mutations in the sarcomere genes. These help muscle contraction, and if they have any type of defect, they can cause cardiomyopathies. The cardiomyopathy most often associated with these mutations is hypertrophic cardiomyopathy, which is characterized by thickened myocardium. Although this is visible in adult hearts, infants who have died from SIDS have had some such mutations in sarcomere genes, but their hearts did not show thickening 64.

The same gene may have different mutations causing different cardiac pathologies. This occurs with the *SCN5A* gene, which when mutated can lead to not only LQTS but also Brudaga syndrome (BrS). Like LQTS, BrS is caused by a mutation in the *SCN5A* gene, which encodes a sodium channel 67. Sodium channels are proteins that let sodium cross cell membranes, thus generating the initial impulse of the cardiac action potential, which is responsible for the functioning of the heart muscle. If *SCN5A* is mutated, cardiac conduction will fail. If there is any such failure, it can be seen in the ST segment of the electrocardiogram, which segment reflects the electrical recovery of the cells. If it is longer than usual, this indicates electrical failure. ST prolongation can be caused by different things, that is, BrS can affect different heart regions through which the electrical impulse passes. One of these regions, which is the most often affected area in children, is the atrial region of the myocardium. If the sinus node, which is a region of cells of the heart that helps the heart rhythm, fails, this can cause arrhythmias or bradycardias 67. Infants can sometimes present with sinus node failure in combination with ventricular arrhythmias. Although atrial disease is the most common manifestation of BrS, BrS can also manifest as a disease of the conduction system, in which there is a loss of conductive function 68.

### c. Alterations in metabolism

Some genetic mutations can lead to failures in the heart system, but other mutations affect other systems, such as metabolism itself. According to previous works 69, metabolic disorders may account for 1% to 2% of SIDS cases. Some genetic dysfunctions of metabolism affect the way glycogen is stored and used, which is known as glycogenosis. This pathology causes fatty changes in the liver, or there are problems in the oxidation of fats caused by the defect or absence of certain enzymes. Among these enzymes, the medium-chain acyl-coenzyme A dehydrogenase can present defects that cause a disorder known as medium-chain acyl-CoA deficiency. This deficiency can cause hypoglycemia: the levels of glucose in the blood decrease in a dangerous way, which can become lethal 70. This disease is caused by a mutation of the *ACADM* gene, which causes too little of this enzyme to be produced, leading to damage that can be fatal to the infant 71. Similarly, the *PCK1* gene, which encodes the enzyme phosphoenolpyruvate carboxykinase, involved in the process of gluconeogenesis, is also involved in metabolic disorders. In addition to causing fatal hypoglycemia, it can cause lactic acid accumulation in the blood 72. Although these pathologies can be treated, the infant might not survive because there have been no symptoms or because the episodes have been too aggressive and continuous, which can happen even if the child is monitored 70. Current studies recommend exhaustively analyzing the levels of acylcarnitines and amino acids extracted from drops of dried blood, since they could be implicated in SIDS 73.

### d. Infections and immune dysfunction

Infections and immune dysfunction are thought to be major pathogenic mechanisms involved in SIDS pathogenesis. Inefficient inflammatory responses against pathogens might explain the relationship between both factors and SIDS mortality, although different causes of this intricate relationship are starting to be elucidated 74.

Immune dysfunction in SIDS has been studied mainly from the perspective of dysregulation of the balance of different pro-inflammatory and anti-inflammatory cytokines 75. Fundamentally, the role of interleukin 1 (IL-1) and, more specifically, IL-1α have been studied. IL-1α is encoded by the *IL1A* gene. Some SIDS infants have had two specific polymorphisms in this gene, and a specific polymorphism of the gene that encodes the IL-1 receptor antagonist (IL-1RA) has been described 70. Similarly, polymorphisms in genes that encode other pro- and anti-inflammatory cytokines, such as IL-6, IL-10, and tumor necrosis factor-alpha (TNF-α), have also been detected 74. These polymorphisms affect the immune response, causing an imbalance in the amount of pro-inflammatory cytokines concerning the anti-inflammatory ones, generating an excessive response to infection, a phenomenon that is associated with SIDS. Simultaneously, in a recent study, Qu et al. 76 evaluated 27 cytokines, chemokines, and growth factors in protein lysates of lungs derived from 29 SIDS cases and compared them with 15 deceased neonates by other causes, obtaining some interesting conclusions. On the one hand, they observed that the chemokine (C-C motif) ligand 5 (CCL5) was elevated in SIDS cases with mild upper airway infections when compared to those without evidence of infection. Besides, a downregulation of IL-1RA, IL-7, IL-13, and granulocyte-colony stimulating factor (G-CSF) was observed in cases of SIDS. They concluded that SIDS may be caused by a variety of factors, including a compromised immune system, an inadequate immunological response to respiratory infections, and an immune response influenced by a Th1/Th2 imbalance 76.

Studies have also established a relationship between the immune response in the laryngeal mucosa and the CNS in SIDS. Some SIDS victims with evidence of mild infection showed elevated levels of IL-6 in the cerebrospinal fluid, as well as a greater number of leukocytes expressing immunoglobulin A (IgA) in the laryngeal mucosa. Still others have had higher expression of the major leukocyte histocompatibility antigen HLA-DR in the glandular epithelium 77. Similar results have been obtained in other organs, such as the trachea or the intestinal mucosa, where the numbers of IgA- and IgM-producing cells have also been increased 78. Ferrante et al. 79 analyzed brain, heart, and liver tissues from 15 SIDS cases and 15 controls using an Illumina whole genome gene expression DASL HT assay. They found significant alterations in 17 genes in neonates with SIDS, being 3 of them tightly linked to the immune system. These changes were the downregulation of MyD88 in tissue from SIDS brains and the downregulation of the genes encoding CCL3 and UNC13 in the liver, supporting that aberrant immune responses might be implicated in the development of SIDS.

The existence of a wide variety of pathogens potentially related to SIDS and immune dysfunction has been evidenced, including enteric bacteria (such as *Staphylococcus aureus*, *Escherichia coli*, and bacteria of the genus Clostridium), respiratory viruses, enteroviruses, and even certain fungi 80. The presence of these infectious agents, together with the aforementioned polymorphisms, appears to have a potential role in the immune dysfunction associated with SIDS, although the causal role of any of these pathogens in SIDS has not been demonstrated 81. Likewise, previous works have suggested a potential pathogenic role of bacterial toxins in SIDS, as the presence of these toxins in sera and tissues from SIDS cases has also been defined 82-85. Indeed, past works have demonstrated that lethal levels of toxins are present in SIDS sera and that they could be neutralized by normal immunoglobulins 86. It seems that different toxins could be exerting a synergic effect in this lethality 87, highlighting the need to consider multiple factors and interactions in the complex dynamics leading to SIDS. In addition, viruses may trigger toxin pathogenicity and exacerbate the effects of bacterial infections and toxins. The relevance of respiratory viruses in SIDS seems to be supported by a considerable amount of evidence 88-91. The literature recognizes that sleeping prone with or without upper respiratory viral infection stimulates the pooling of nasopharyngeal secretions and the enhancement of nasopharyngeal colonization by toxigenic *Staphylococcus aureus* and *Escherichia coli* 45. Also, the prone position increases nasopharyngeal temperature into ranges that favor bacterial toxin production 92. According to past works, the elevated risk of SIDS when sleeping prone is increased by each of four factors: the use of natural-fiber mattresses, swaddling, recent infections, and the use of heating in bedrooms 93. In other words, epidemiological data supports that having respiratory infections is strongly associated with the increased risk of suffering from SIDS when sleeping prone, suggesting the relevance of this factor 94. Similarly, myocardial infections produced by different types of viruses have been proposed as a critical approach to consider to understand the etiopathogenesis of SIDS 95, although other studies show that the frequency and relevance of viral infections are indeed low, and it needs to be further explored 96. Overall and as previously commented, rather than a cause itself as proposed in the triple risk hypothesis, sleeping in a prone position might be potentially linked to infections and lethal toxins, explaining its role in SIDS.

The picture is further complicated not only by the infectious processes but also by the intestinal microbiota itself, which may be responsible for the alterations that occur in the immune system. Among the most significant findings about the microbiota, infants with SIDS tend to show specific differences in some species, such as *Clostridium difficile*, *Clostridium innocuum*, and *Bacteroides thetaiotaomicron* 34*.* Similarly, infants with SIDS tend to have greater *Staphylococcus aureus* colonization and to have high levels of staphylococcal enterotoxin 97. These have more often been found in those babies found in the prone position, which might be related to ingestion or inhalation of bacteria from contaminated surfaces, helping to explain the immune dysfunction 34.

Finally, although more studies on this topic are needed, some authors have suggested a possible relationship between autoimmune phenomena and SIDS, establishing a possible relationship between these phenomena and cardiorespiratory dysfunction associated with pituitary adenylate cyclase activator polypeptide and vasoactive intestinal peptide in the brain 98. Thus, the potential role of immune system dysfunction and SIDS, mainly in its relationships with infectious agents and microorganisms, represents a key point in these studies.

### e. Similarities to epilepsy

Another possible factor in deaths from SIDS is that the infant has similar mutations as an infant with epilepsy. Infants diagnosed with epilepsy face a higher risk of sudden unexpected death in epilepsy 99. In the context of monogenic models, the failure of inhibitory synaptic drive is highlighted as a significant pathogenic stage, with genes like the serotonin receptor 5-HT2C playing a role in suppressing forebrain and brainstem networks, potentially leading to deadly induced audiogenic seizures 100 101 102 The SCN1A gene, commonly mutated in both epilepsy and SIDS infants, presents variants causing sodium channel dysfunctions that affect nerve and muscle action potential transmission, sometimes leading to death through seizures or without any epilepsy-like symptoms 103. CO2 elevation and impaired CO2 arousal mechanisms are proposed mechanisms of death in both conditions, though studies note differences, such as hippocampal abnormalities being more marked in other types of sudden unexpected deaths 104 105 Understanding the various genes and factors involved in sudden unexpected death in epilepsy is crucial for preventing SIDS and related unexpected deaths.

### f. Biomarkers of interest

Biomarkers are biological substances that can be found in the body and that help to determine a person's physiological state. Thus, depending on their application, biomarkers can be diagnostic (if they are characteristic of a certain pathology or condition), prognostic (if they help to establish the evolution of the patient), or predictive (if they can be used to predict how the body will react to a certain treatment or pathology) 106. Many of the biomarkers used in SIDS are evaluated postmortem, such as the various genes and proteins mentioned throughout this section. One of the main challenges is finding biomarkers that can distinguish infants at risk of SIDS to prevent it. In this sense, a recent study suggests the relevance of butyrylcholinesterase as a potential biomarker of great predictive value in these infants 107.

Butyrylcholinesterase is an enzyme of the cholinergic system that regulates many of the effects mediated by the ANS. This enzyme, together with the enzyme acetylcholinesterase, hydrolyses acetylcholine, the main neurotransmitter of the ANS. Once the acetylcholine is hydrolyzed, it is broken into acetate and choline. This choline molecule is reincorporated into the neurons so that it can be used again. Therefore, these enzymes are essential for the proper functioning of the cholinergic system and therefore of the ANS 108. From the study of butyrylcholinesterase measured in drops of dried blood taken 2 or 3 days after birth, in infants who died of SIDS, the blood had reduced levels of butyrylcholinesterase in comparison with other infants who had died of a known cause or with healthy controls who had similar characteristics to the deceased 107. A low amount of this enzyme implies dysfunctions in the cholinergic system: As there is less choline, there will not be adequate amounts of choline for ANS processes. This can impair respiratory function, which would leave the child in a vulnerable situation, being more susceptible to SIDS. Despite the promising results, researchers have been cautious about translating this biomarker to clinical treatment, since there are some important questions to solve: mainly, if the results obtained in these samples are representative of what may be happening in the neural synapses, the relationship that this reduction has with acetylcholinesterase, and even if these results will be replicated in other cohorts 109. However, one of the most important concerns to consider from this type of study is the relevance of providing and preserve samples from babies deceased by SIDS, acknowledging the necessity for future screening to operate within current systems for sample collection and availability 110.

**Figure 2:**
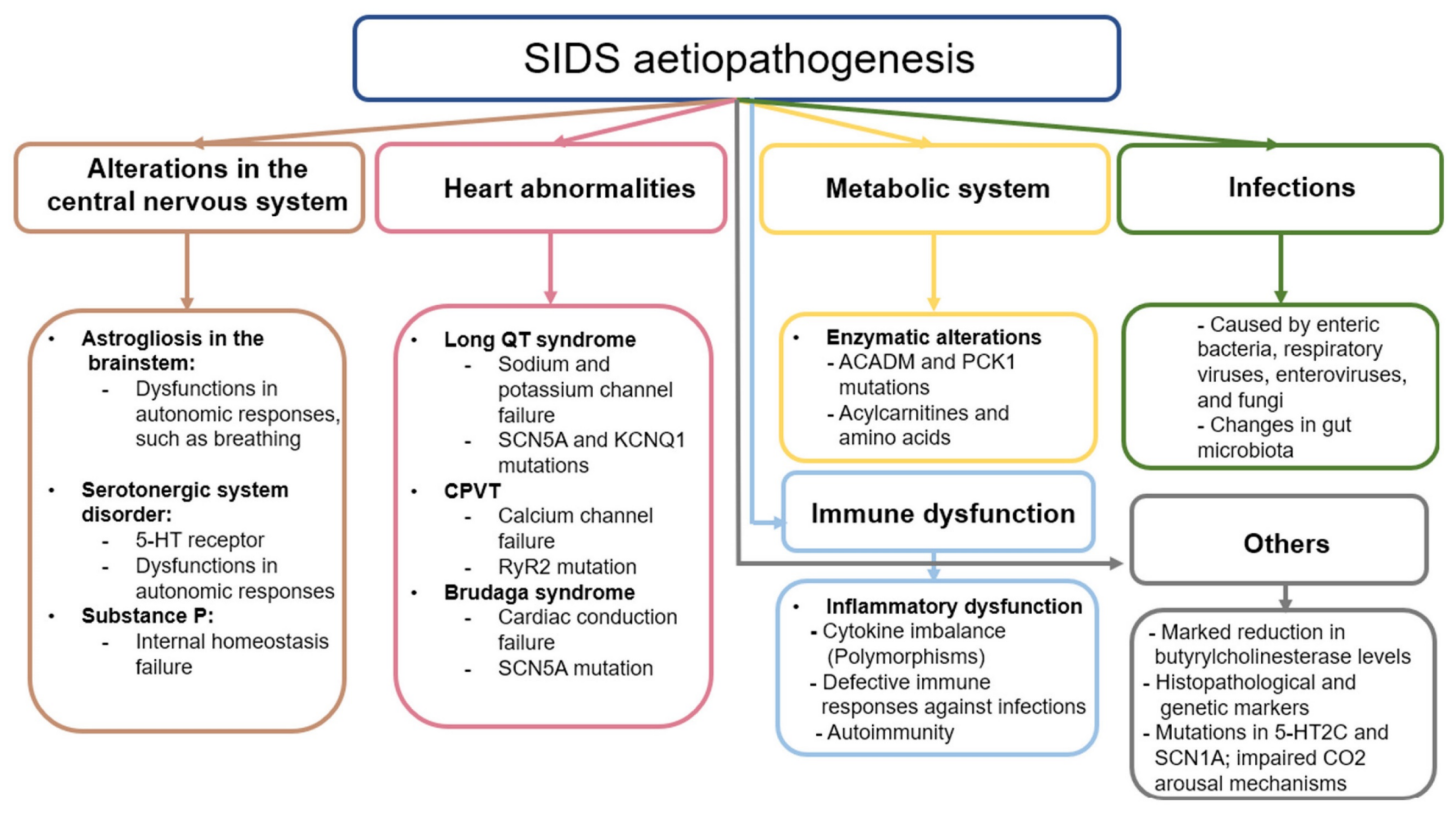
Schematic representation of the etiopathogenesis of infants with SIDS.

Figure 2 shows the current knowledge of the aetiopathogenesis of SIDS, highlighting once again the need for future studies that delve into the causes and mechanisms underlying this tragedy.

## 6. Future directions

Experts propose various approaches to Sudden Infant Death Syndrome (SIDS) 111 encompassing optimization of entity characterization, exploration of etiopathogenesis, advocacy for prevention measures, and the enhancement of health workers' role in supporting affected parents. The first approach emphasizes the imperative need for precise identification and classification of SIDS, with models like that proposed by Garstang et al. critical for recognition 112. Standardized screening processes, application of percentile graphics, and addressing potential asphyxia aid in improving identification 111. Molecular understanding of intrinsic risk factors involves addressing ethical concerns, the lack of progress in identifying causes, and utilizing advanced techniques like next-generation DNA sequencing (NGS) for genetic diagnosis 14,45,113,114. NGS techniques, including short-read and long-read sequencing, play a fundamental role in detecting genetic defects related to SIDS 115. During the fetal stage, tests such as ultrasonography, electrocardiography, echocardiography, and magnetocardiography can be performed to monitor heart development and detect potential issues 116-120. Most diagnostic tests are done on the infant or fetus, but some techniques can also be applied to parents as well 121. Psychological and humanitarian measures for parents dealing with SIDS involve recognizing the difficult process of loss, addressing guilt, and providing continuous support to prevent self-harm or suicidal ideation 122-125. For this reason, it is important to offer a follow-up to help parents overcome the intense pain and prevent them from being pushed toward suicidal actions.

## 7. Conclusions

As has been demonstrated throughout this article, even though it become rarer, SIDS continues to be a type of infant death that is too common among infants in different regions of the world, such as the United States and Spain. Although it is a sudden and unexplained death, several studies have been carried out in recent years to try to explain. These have found risk factors that predispose the infant to suffer this type of death. They pertain to different organs and systems, such as the heart, the CNS, the immune system, and metabolism, and they are related to epilepsy and specific biomarkers. Most of them have a genetic component, so technological advances in sequencing, together with other techniques, have achieved an early recognition of infants vulnerable to SIDS. Future studies should delve into the mechanisms underlying this condition. All of this is expected to help parents find an explanation for the death of their infant, lessening the harsh process of mourning that this event entails and avoiding possible mental or physical problems, such as suicide.

## Figures and Tables

**Table 1:** Subcategories of SIDS according to 2.

**Category Ia Classic features with complete investigation**	Infant deaths that meet the requirements of the general definition and also all of the following requirements: **Clinical:** More than 21 days and less than 9 months of age; Normal clinical history, including term pregnancy (gestational age of >37 weeks).; Normal growth and development; No similar deaths among siblings, close genetic relatives (uncles, aunts, or first-degree cousins), or other infants in the custody of the same caregiver. **Circumstances of death:** Investigation of the various scenes where incidents leading to death might have occurred and determination that they do not explain the death and determination that they do not provide an explanation for death found in a safe sleeping environment with no evidence of accidental death. **Autopsy:** Absence of potentially fatal pathologic findings; Minor respiratory system inflammatory infiltrates are acceptable; intrathoracic petechial hemorrhage is a supportive but not obligatory or diagnostic finding; No evidence of unexplained trauma, abuse, neglect, or unintentional injury; No evidence of substantial thymic stress effect (thymic weight less than 15 g and/or moderate/severe cortical lymphocyte depletion); Occasional “starry sky” macrophages or minor cortical depletion is acceptable; Negative results of toxicologic, microbiologic, radiologic, vitreous chemistry, and metabolic screening studies
**Category Ib Classic features with an incomplete investigation**	An infant death that meets the requirements of the general definition and also meets all of the above criteria for Category IA except that: investigation of the various scenes where incidents leading to death may have occurred was not performed, and/or one or more of the following analyses was not performed: toxicology, microbiology, radiology, vitreous chemistry, and metabolic screening.
**Category II**	An infant death that meets Category I criteria except for one or more of the following: **Clinical:** Age range — outside Category IA or IB, i.e. 0 to 21 days or 270 to 365 days; similar deaths of siblings, close relatives, or other infants in the custody of the same caregiver that are not considered suspicious for infanticide or for recognized genetic disorders; neonatal and perinatal conditions (e.g. those resulting from preterm birth) that have resolved by the time of death. **Circumstances of death:** Mechanical asphyxia or suffocation by overlaying not determined with certainty. **Autopsy:** Abnormal growth and development not thought to have contributed to death; marked inflammatory changes or abnormalities not sufficient to be unequivocal causes of death.
**USID: Unclassified sudden infant deaths**	This includes deaths that did not meet the criteria for Category l or II SIDS, but where alternative diagnoses of natural or unnatural conditions were equivocal (including cases where autopsies have not been performed).

**Table 2:** Recommendations for the prevention of SIDS.

Preventive measures	Evidence level^1^
Supine position	A
Overwatch "Tummy Time"	B
Firm sleeping surface	A
Sleeping without objects around	A
Do not sleep with someone on the same surface	A
Sleeping in the same room on another surface	A
Do not wrap the infant too much	A
Use pacifiers	A
To breastfeed	A
Adequate control of pregnancy	A
Do not take psychoactive substances during pregnancy	A
Avoid exposing the infant to tobacco smoke	A
Avoid wrapping the infant with too many layers	C
Postnatal and pediatric follow-up	A
Comply with the vaccination plan	A

^1^ Grade of recommendation of SIDS prevention measures according to the AAP. The USPSTF states that “A” is for recommendations that seem to be more supported by scientific evidence; “B” is similar to “A”, but the observed benefits appear to be more moderate; whereas the recommendations that are classified as “C” are those that scientific evidence have not drawn significant benefits.
